# Exploring the Spectrum of Comorbidities Associated with Primary Aldosteronism: Insights from a Large Real-World Case-Control Study

**DOI:** 10.3390/biomedicines12112479

**Published:** 2024-10-29

**Authors:** Andreas Krieg, Sarah Krieg, Andreas Heuser, Ulrich Laverenz, Valentin-Alin Istrate, Matthias Schott, Karel Kostev

**Affiliations:** 1 Department of General and Visceral Surgery, Thoracic Surgery and Proctology, University Hospital Herford, Medical Campus OWL, Ruhr University Bochum, 32049 Herford, Germany; andreas.heuser@rub.de (A.H.); ulrich.laverenz@klinikum-herford.de (U.L.); valentin-alin.istrate@klinikum-herford.de (V.-A.I.); 2 Department of Inclusive Medicine, University Hospital Ostwestfalen-Lippe, Bielefeld University, 33617 Bielefeld, Germany; sarah.krieg@mara.de; 3 Division for Specific Endocrinology, Heinrich-Heine-University and University Hospital Duesseldorf, 40225 Duesseldorf, Germany; matthias.schott@med.uni-duesseldorf.de; 4 Epidemiology, IQVIA, 60549 Frankfurt, Germany

**Keywords:** Conn syndrome, primary aldosteronism, adrenal gland, hypertension, hypokalemia, hepatic steatosis, gout, chronic kidney disease, diabetes mellitus

## Abstract

**Background**: Primary aldosteronism (PA) is a common cause of endocrine hypertension, characterized by excessive aldosterone secretion leading to hypertension, hypokalemia, and metabolic alkalosis. While historically diagnosed based on this classic triad of symptoms, current understanding reveals a more nuanced presentation. This study aimed to investigate the prevalence of PA-associated diseases in a large German population. **Methods**: Medical records from the IQVIA^TM^ Disease Analyzer database were analyzed retrospectively. PA patients (n = 860) were matched with non-PA individuals (n = 4300) by age and sex. Associations between PA and predefined chronic diseases were examined using multivariable logistic regression. **Results**: PA was significantly associated with hypokalemia (7.8% vs. 1.6%, odds ratio (OR): 3.45; 95% confidence intervals (CIs): 2.41–4.96), hypertension (56.1% vs. 28.5%; OR: 2.37; 95% CIs: 2.00–2.81), hepatic steatosis (11.3% vs. 3.0%; OR: 1.85; 95% CIs: 1.34–2.57), gout (8.3% vs. 2.2%; OR: 1.64; 95% CIs: 1.15–2.35), chronic kidney disease (6.3% vs. 2.2%; OR: 1.59; 95% CIs: 1.10–2.31), diabetes mellitus not otherwise specified (7.9% vs. 2.9%; OR: 1.49; 95% CIs: 1.06–2.09), obesity (13.5% vs. 5.1%; OR: 1.38; 95% CIs: 1.05–1.82), and depression (14.8% vs. 6.2%; OR: 1.37; 95% CIs: 1.07–1.77). **Conclusions**: While the study design had limitations, including reliance on ICD codes for diagnosis, these findings underscore the critical need for early detection and personalized management strategies for PA to reduce associated risks and improve patient outcomes.

## 1. Introduction

In 1956, Jerome Conn reported the case of a woman with a disorder he termed primary aldosteronism (PA), which included generalized weakness and transient paralysis, muscle spasms, hypertension, and hypokalemia. The term “primary” was used to distinguish this form of hyperaldosteronism from secondary forms that can occur in intravascular volume depletion, congestive heart failure, and cirrhosis [1].

PA is the most common cause of endocrine hypertension and occurs in approximately 4–13% of hypertensive patients in general practice [2,3,4]. PA is characterized by an autonomous and uncontrolled aldosterone secretion [5]. The resulting over-activation of the mineralocorticoid receptor leads to volume retention, hypertension, hypokalemia, and metabolic alkalosis. However, it is now known that hypokalemia occurs in only 46% of patients and the classic triad of arterial hypertension, hypokalemia, and metabolic alkalosis occurs in only a small proportion of patients, most of whom are severely affected [6,7]. The cause of uncontrolled aldosterone secretion is bilateral adrenal hyperplasia in two thirds of cases and unilateral adrenal adenoma in one third [5].

The subtyping between unilateral and bilateral disease is of particular importance in PA. Since a small adenoma of the adrenal cortex is typically responsible for the hypersecretion of aldosterone in unilateral disease, the treatment of choice for unilateral PA is minimally invasive (e.g., retroperitoneoscopic, laparoscopic) partial or total adrenalectomy [8,9,10]. In contrast, patients with proven bilateral hyperaldosteronism or those who are not candidates for surgery usually require lifelong therapy with mineralocorticoid receptor antagonists (MRAs) like spironolactone or eplerenone [8]. In many cases, additional antihypertensive therapy is required to adequately control blood pressure. The treatment with MRAs can be complemented or substituted with agents such as amiloride, which directly inhibit the epithelial sodium channel [11]. Furthermore, thiazide diuretics and calcium channel blockers can be combined with treatment with MRAs or amiloride. However, a low-salt diet and close monitoring of blood pressure and serum electrolytes are also key elements in the treatment plan [11].

Patients with PA have an approximately 3 to 12 times higher risk of cerebrovascular (e.g., stroke), cardiovascular (e.g., atrial fibrillation, coronary artery disease, left ventricular hypertrophy, heart failure) events, or chronic kidney disease compared to patients with essential hypertension [12,13,14,15,16,17,18,19]. Consequently, recommendations from various national and international medical societies, including the American College of Cardiology/American Heart Association [20] and the Endocrine Society [8], recommend that patients with refractory hypertension and hypertension with hypokalemia be evaluated for the presence of PA by measuring plasma aldosterone–renin activity or concentration. Additionally, metabolic disorders like type 2 diabetes and metabolic syndrome [19,21,22], psychiatric changes [23], and obstructive sleep apnea syndrome (OSAS) [24] are increasingly observed in patients with PA. Therefore, the Endocrine Society has already recommended that patients with OSAS should also be evaluated for the presence of PA [8].

Additional knowledge about the association of certain diseases with PA could help to modify screening strategies and provide a basis for earlier diagnosis, treatment, and prevention of PA sequelae in the general population. Thus, this study aimed to investigate the prevalence of potential PA-associated diseases compared with those observed in a control group of patients without PA and patients with hypertension but without PA in a large real-world German population.

## 2. Materials and Methods

### 2.1. Database

For this study, medical records (baseline demographics and diagnoses) from the IQVIA^TM^ Disease Analyzer database were used. Data from this repository were obtained directly and anonymously from electronically stored data systems used by primary-care physicians and specialists in private practice in Germany [25]. The database comprises approximately 3000 physicians selected by panel design according to predefined strata including specialty, federal state, community size category, and physician age. While previous research has shown the database to be representative for the country [25], it has been widely used in several studies of epidemiology including comorbidities [26,27].

### 2.2. Study Population

**Figure 1:**
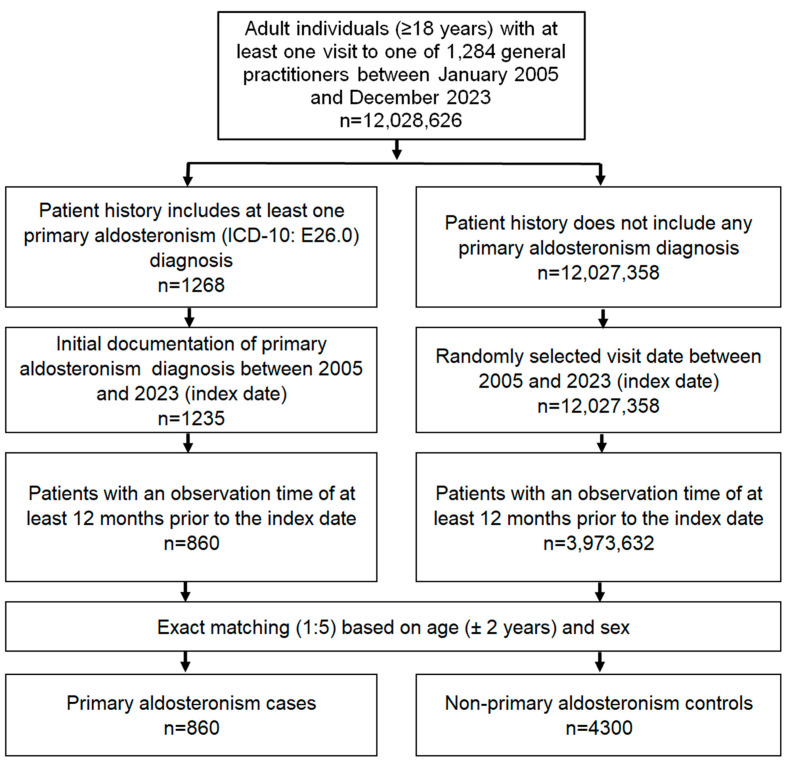
Stepwise selection of eligible study patients.

Our retrospective case-control study enrolled outpatients aged ≥18 years who had a first documented diagnosis of PA (ICD-10: E26.0) by a general practitioner (GP) between January 2015 and December 2023 (index date). Of note, this PA diagnosis did not necessarily have to be made by the GP, but could also be made by a specialist or in a hospital and then documented by the GP. Importantly, we must acknowledge that ICD codes were assigned by local protocols and may not have been standardized across the study. Only patients with data available from at least 12 months before the index date were included in the analysis. This inclusion criterion was necessary to ensure that PA was not documented prior to the index date and to allow analysis of diagnoses documented within 12 months of PA diagnosis. PA patients were matched 1:5 with non-PA individuals for age and sex. The index date for non-PA individuals was a random physician visit between January 2015 and December 2023 (Figure 1).

### 2.3. Study Outcomes and Covariates

The study outcomes were the prevalence of various disorders documented within the 365 days prior to the index date and the associations between these disorders and PA. All diagnoses or diagnosis classes occurring in at least 5% of the study patients were analyzed. These disorders included nontoxic goiter (ICD-10: E04), diabetes mellitus type 2 (ICD-10: E11), diabetes mellitus not otherwise specified (ICD-10: E14), obesity (ICD-10: E66), disorders of lipoprotein metabolism (ICD-10: E78), hypokalemia (ICD-10: E87.6), hypertension (ICD-10: I10), chronic ischemic heart disease (ICD-10: I25), depression (ICD-10: F32, F33), sleep disorders (ICD-10: G47), gastritis and duodenitis (ICD-10: K29), non-alcoholic fatty liver disease (NAFLD, ICD-10: K76.0), gout (ICD-10: M10), back pain (ICD-10: M54), and chronic kidney disease (ICD-10: N18). Additionally, the proportion of patients with prescriptions for spironolactone within the 365 days prior to the index date was evaluated.

### 2.4. Statistical Analyses

The prevalence of defined disorders was calculated for individuals with and without PA. Multivariable logistic regression models were used with all study disorders as dependent variables and PA as an outcome variable. Additionally, spironolactone was also investigated as a dependent variable. *p* values < 0.05 were considered statistically significant. Analyses were performed with SAS version 9.4 (SAS Institute, Cary, NC, USA).

### 2.5. Sensitivity Analyses

For sensitivity analysis, PA patients were matched 1:5 with non-PA individuals with hypertension diagnosis by age and sex. Both descriptive and regression analyses were repeated for new matched pairs.

## 3. Results

### 3.1. Baseline Characteristics of the Study Patients

Following a 1:5 matching, we enrolled 860 individuals with PA and 4300 without PA in our study. Baseline characteristics of the study patients are summarized in Table 1. In the entire study population, 51.1% of patients were female. The mean age in the PA group was 60.9 (SD: 14.5) years and 61.1 (SD: 14.7) years in the non-PA group.

### 3.2. Prevalence of Predefined Disorders

**Figure 2:**
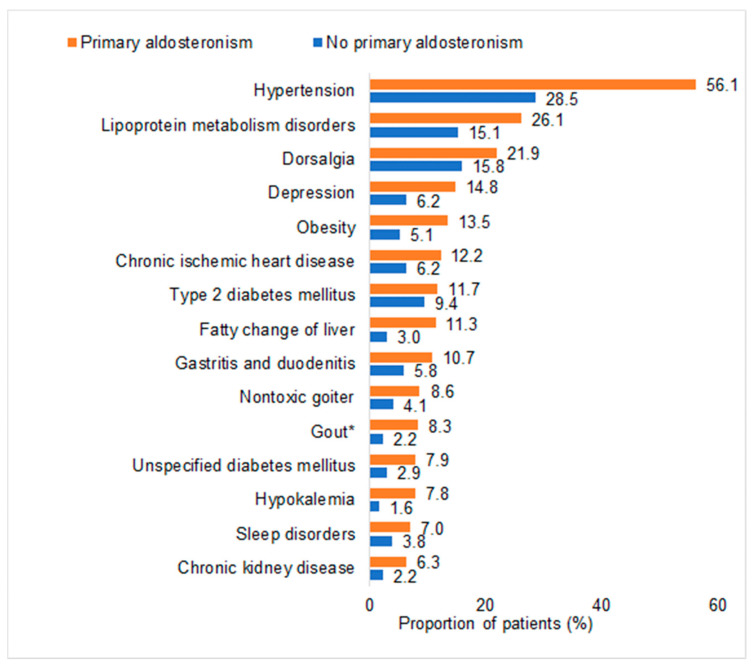
Prevalence of different disorders documented within 12 months prior to the index date. * A direct association between PA and gout could not be found in patients who were not treated with spironolactone.

Among PA patients, hypertension was the disorder with the highest prevalence (56.1%), followed by lipoprotein metabolism disorders (26.1%), dorsalgia (21.9%), depression (14.8%), and obesity (13.5%). Hypertension (28.5%), lipoprotein metabolism disorders (15.1%), and dorsalgia (15.8%) were also the most common disorders in non-PA individuals, but the prevalence rates of these disorders were markedly lower in controls than in PA cases (Figure 2). In addition, 18.5% of the patients with PA and 2.6% of the controls were treated with spironolactone.

### 3.3. Association of PA with Predefined Disorders

Using multivariable logistic regression, we identified a significant positive association between PA and eight conditions: hypokalemia (7.8% vs. 1.6%, odds ratio (OR): 3.45; 95% confidence intervals (CIs): 2.41–4.96), hypertension (56.1% vs. 28.5%; OR: 2.37; 95% CIs: 2.00–2.81), hepatic steatosis (11.3% vs. 3.0%; OR: 1.85; 95% CIs: 1.34–2.57), gout (8.3% vs. 2.2%; OR: 1.64; 95% CIs: 1.15–2.35), chronic kidney disease (6.3% vs. 2.2%; OR: 1.59; 95% CIs: 1.10–2.31), diabetes mellitus not otherwise specified (7.9% vs. 2.9%; OR: 1.49; 95% CIs: 1.06–2.09), obesity (13.5% vs. 5.1%; OR: 1.38; 95% CIs: 1.05–1.82), and depression (14.8% vs. 6.2%; OR: 1.37; 95% CIs: 1.07–1.77) (Table 2). Furthermore, spironolactone use was strongly positively associated with PA (OR: 7.46; 95% CIs: 5.54–10.05). To investigate whether the association between PA and gout was primarily due to PA and not to spironolactone therapy, only patients not receiving spironolactone therapy were examined for this association. However, this regression analysis did not reveal any direct association between PA and gout (OR: 1.23; 95% CIs: 0.74–2.03; *p* = 0.429).

### 3.4. Sensitivity Analyses

**Figure 3:**
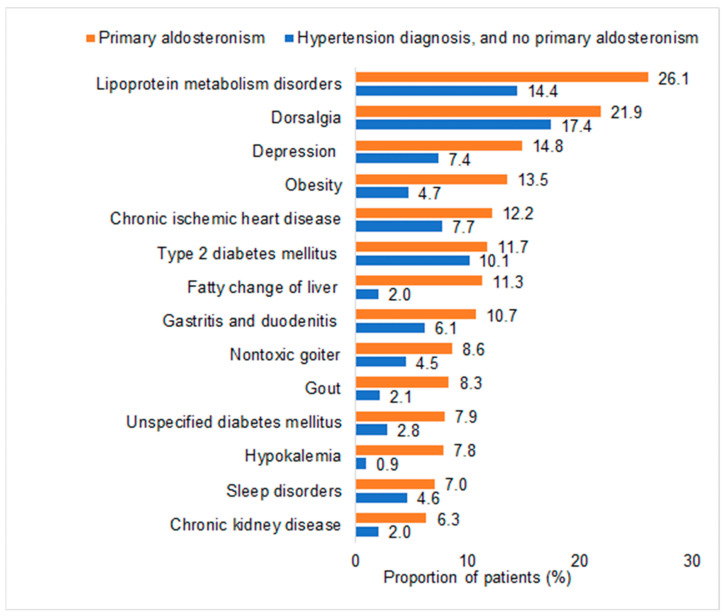
Prevalence of different disorders documented within 12 months prior to the index date (individuals with PA versus individuals with hypertension and without PA).

Figure 3 shows the prevalence of chronic disorders among PA patients and age- and sex-matched non-PA patients with hypertension.

In regression analyses, a significant positive association was observed between PA and five disorders: hypokalemia (OR: 14.32; 95% CIs: 9.50–21.56), fatty change of liver (OR: 2.23; 95% CIs: 1.46–3.42), chronic kidney disease (OR: 2.73; 95% CIs: 1.77–4.23), obesity (OR: 5.07; 95% CIs: 3.94–6.54), and depression (OR: 1.99; 95% CIs: 1.52–2.59). Diabetes mellitus type 2 was negatively associated with PA (OR: 0.41; 95% CIs: 0.29–0.57) (Table 3). Note that only 3.0% of the controls were treated with spironolactone and spironolactone use was strongly positively associated with PA (OR: 9.49; 95% CIs: 7.14–12.62).

## 4. Discussion

PA, caused by autonomous hypersecretion of aldosterone from the adrenal cortex, occurs in about 4–13% of hypertensive patients in general practice [2,3,4] and may be associated with hypokalemia, although this is seen in only about 46% of patients [7]. The aim of our study was to explore, in a real-world dataset, which chronic diseases were present prior to the diagnosis of PA and may therefore be strongly associated with PA. At this point, we must emphasize that there are other registries, such as the German Conn Registry [28] and the Spanish Primary Aldosteronism Registry of the Spanish Society of Endocrinology and Nutrition (SEEN) (SPAIN-ALDO Registry) [29]. Importantly, studies using data from these registries showed many of the associations we found in our dataset [30,31,32,33,34]. However, in contrast to our database, the German Conn Registry includes centers that specialize in patients with primary aldosteronism. Therefore, the purpose of our study was to provide an overview of patients treated by general practitioners rather than specialized centers.

In our study, hypertension and hypokalemia were observed in 56.1% and 7.8% of patients diagnosed with PA, respectively. These relatively low rates may be due to the fact that we could not specify whether the diagnosis was made as part of screening for hypertension or as part of the diagnosis of incidental adrenal tumor. It should be noted that hypertension and hypokalemia are not fundamental manifestations of PA, but, rather, depend on the severity and duration of exposure to renin–autonomous-aldosterone secretion and sodium retention [35]. Although hypertension is a common symptom of primary hyperaldosteronism and occurs in most patients, rare forms of normotensive PA have also been described in the literature [36]. In the context of normotensive PA, it has been postulated that these patients may be in an early stage of the disease, and presenting with mild or non-elevated blood pressure within the normal range [36].

However, this does not explain the relatively high prevalence of PA patients without a diagnosis of arterial hypertension that we observed in our cohort. Therefore, it cannot be excluded that some of the patients included in our study may have been receiving antihypertensive medication at the time of coding the diagnosis, which could have led to misclassification as normotensive. We also cannot exclude the possibility that different reference or cut-off values for hypokalemia and different definitions of blood pressure were used, and our database does not contain information on the method by which blood pressure was measured. Nevertheless, our multivariable analysis showed that both hypertension and hypokalemia were the most commonly associated conditions in patients with PA.

Using aldosterone–renin quotient (ARQ) screening, the prevalence of PA in primary-care patients treated for hypertension is approximately 4–13% [2,3,4]. Unfortunately, even today, many patients are not being screened. In this context, a retrospective cohort study of U.S. veterans with refractory hypertension between 2000 and 2017 found that only 1.6% of patients were screened for the presence of PA [37]. In addition, it is important to note that the rate of screening for the presence of PA was highly dependent on the specialty of the primary-care provider and that screening for the presence of PA was an important determinant of initiation of MRA therapy, and thus, blood-pressure control. In particular, in the study by Cohen et al., the screening rate was lowest in the group of general practitioners [37]. However, years of delay in diagnosis predispose the development of end-organ damage, and it is well known that patients diagnosed with PA have a higher incidence of cardiovascular morbidity and mortality as well as renal morbidity than patients of comparable age or blood pressure [19,38]. The cardiovascular effects of PA are both diverse and substantial, ranging from left ventricular hypertrophy, worsening of systolic and diastolic function, myocardial ischemia, vascular remodeling, arrhythmias, and sudden cardiac death [39]. The pathophysiological mechanisms leading to vascular damage are not limited to hypertension, which alone is a significant problem for the cardiovascular system. In addition, the induction of oxidative stress, endothelial inflammation, and vascular damage leading to fibrosis or podocyte damage plays a role in the pathogenesis of aldosterone-induced cardiac or chronic kidney disease [40,41,42,43]. Although PA patients in our study sample were almost twice as likely to have chronic ischemic heart disease, with a prevalence of 12.2%, this association was not significant in multivariable analysis. In contrast, chronic kidney disease had an almost 3-fold higher prevalence compared with the control group and patients with hypertension and no PA, which was also significant in the multivariable analysis.

To optimize and standardize the early detection, diagnosis, and treatment of PA, the Endocrine Society has developed a guideline [8]. According to these recommendations, patients should be evaluated for the presence of PA based on well-defined anamnestic and diagnostic criteria. These criteria are based on an increased prevalence of PA in defined risk groups, including OSAS. Unfortunately, our data search was unable to look specifically for the ICD-10 code G47.31, which is defined for OSAS. The lack of association with sleep disorders in our population could therefore be explained by the fact that our search for code G47 was only a general and therefore not a very detailed screen for sleep disorders.

The association between PA and depression observed in our study is also intensively discussed in the literature [23]. Numerous studies have already shown that elevated plasma aldosterone concentrations are associated with depression [44,45,46] and that adrenalectomy with normalization of aldosterone levels leads to a reduction in depressive symptoms [47].

In addition, PA is associated with decreased insulin sensitivity and secretion [48,49] and an increased incidence of metabolic syndrome and type 2 diabetes mellitus [19]. We also found a significant association between PA and obesity or unspecific diabetes mellitus (E14), but not with type 2 diabetes mellitus (E11) in our study population. Interestingly, there was a particularly strong association between the presence of NAFLD and PA. Our observations thus confirm studies conducted in much smaller populations [50,51].

Interestingly, patients with PA were 3.8 times and almost 4 times more likely to develop gout than the control group and the group of patients with hypertension but without PA, respectively. To our knowledge, an association between PA and gout has not been described in the literature to date. It is known that therapy with spironolactone, which is also used for PA, can lead to an increase in blood uric acid levels, which in turn can cause gout flares in predisposed patients. In our study population, 18.5% of the patients with PA, but only 2.6% of the controls, were treated with spironolactone. Therefore, it is tempting to speculate that the association we observed between PA and gout may have been affected by spironolactone therapy. To test the hypothesis that there might be a positive association between PA and gout, we excluded patients receiving spironolactone from a further regression analysis, limiting the analyses to patients not using this medication. However, no association was found, suggesting that the observed association between PA and gout may be at least partially explained by spironolactone therapy. Although we were able to confirm the association between PA and numerous chronic diseases in a relatively large patient population, we must point out some limitations, especially in the study design. As all diagnoses were based on ICD codes, we cannot exclude the possibility of misclassification. In addition, we cannot provide data on the validity of the diagnosis of PA, nor can we show which diagnostic tools were used to make the diagnosis of PA. ICD-10 classification does not allow us to differentiate between symptomatic and non-symptomatic diseases. Furthermore, patients identified with PA may represent only a small fraction of the patients in the German healthcare system who truly have PA. This may bias the differences between PA and non-PA individuals in the study with a consequence that PA cases identified are not fully representative of the clinical spectrum of PA. Finally, our database did not allow us to examine the effects of aldosterone antagonists or other medications, although spironolactone treatment was strongly positively associated with PA in our study.

Nevertheless, we believe that our study represents an important contribution to the literature because we were able to perform our investigations on a very large sample. In addition, the PA cases were age- and sex-matched to the control group.

## 5. Conclusions

In contrast to previous studies based on data from highly specialized centers, our study provides an overview of patients with PA and associated comorbidities who were treated by general practitioners. Through retrospective analysis of a large number of outpatients, the study confirmed a significant association between PA and various chronic diseases, including hypertension, hypokalemia, hepatic steatosis, gout, chronic kidney disease, obesity, and depression. Notably, PA patients exhibited a markedly higher likelihood of developing gout compared with the control group. While acknowledging limitations in the study design, such as reliance on ICD codes for diagnosis, the findings emphasize the critical need for early detection and individualized management strategies for PA to mitigate associated risks and improve patient outcomes.

## Figures and Tables

**Table 1:** Age and sex characteristics of study patients with or without primary aldosteronism (PA) prior to and after 1:5 matching.

	Prior to Matching			After Matching		
Variable	Proportion Among Individuals with PA (n, %)	Proportion Among Individuals Without PA (n, %)	*p*-Value	Proportion Among Individuals with PA (n, %)	Proportion Among Individuals Without PA (n, %)	*p*-Value
N	860	3,973,632		860	4300	
Sex: female	439 (51.1)	2,102,813 (52.9)	0.271	439 (51.1)	2195 (51.1)	1.000
Sex: male	421 (48.9)	1,870,819 (47.1)		421 (48.9)	2105 (48.9)	
Age (mean, SD)	60.9 (14.5)	51.2 (20.0)	<0.001	60.9 (14.5)	61.1 (14.7)	0.685
Age ≤ 40	78 (9.1)	1,336,163 (33.6)	<0.001	78 (9.1)	406 (9.4)	0.945
Age 41–50	120 (14.0)	577,794 (14.5)		120 (14.0)	608 (14.1)	
Age 51–60	209 (24.3)	684,383 (17.2)		209 (24.3)	1030 (24.0)	
Age 61–70	208 (24.2)	569,630 (14.3)		208 (24.2)	1032 (24.0)	
Age > 70	245 (28.5)	805,662 (20.3)		245 (28.5)	1224 (28.5)	

**Table 2:** Association between primary aldosteronism (PA) and several disorders in individuals followed in general practices in Germany (reference group: all individuals without PA). * A direct association between PA and gout could not be found in patients who were not treated with spironolactone.

Diagnosis (ICD-10 Codes)	Odds Ratio for PA (95% CIs) ^#^	*p*-Value
Hypokalemia (E87.6)	3.45 (2.41–4.96)	<0.001
Hypertension (I10)	2.37 (2.00–2.81)	<0.001
Fatty change of liver (K76.0)	1.85 (1.34–2.57)	<0.001
Gout (M10) *	1.64 (1.15–2.35)	0.007
Chronic kidney disease (N18)	1.59 (1.10–2.31)	0.014
Unspecified diabetes mellitus (E14)	1.49 (1.06–2.09)	0.021
Obesity (E66)	1.38 (1.05–1.82)	0.023
Depression (F32, F33)	1.37 (1.07–1.77)	0.014
Nontoxic goiter (E04)	1.30 (0.96–1.75)	0.088
Disorders of lipoprotein metabolism (E78)	1.20 (0.97–1.49)	0.091
Dorsalgia (M54)	1.18 (0.98–1.43)	0.088
Gastritis and duodenitis (K29)	1.13 (0.87–1.49)	0.362
Sleep disorders (G47)	1.11 (0.80–1.54)	0.516
Chronic ischemic heart disease (I25)	1.08 (0.83–1.41)	0.560
Type 2 diabetes mellitus (E11)	0.79 (0.61–1.01)	0.063

**^#^** Multivariate logistic regression adjusted for all diagnoses listed in the table; *p* < 0.05 is considered statistically significant.

**Table 3:** Association between PA and several disorders in individuals followed in general practices in Germany (reference group: individuals with hypertension and without PA).

Diagnosis (ICD-10 Codes)	Odds Ratio for PA (95% CIs) *	*p*-Value
Hypokalemia (E87.6)	14.32 (9.50–21.56)	<0.001
Fatty change of liver (K76.0)	2.23 (1.46–3.42)	<0.001
Gout (M10)	1.56 (0.98–2.49)	0.056
Chronic kidney disease (N18)	2.73 (1.77–4.23)	<0.001
Unspecified diabetes mellitus (E14)	1.29 (0.85–1.95)	0.070
Obesity (E66)	5.07 (3.94–6.54)	<0.001
Depression (F32, F33)	1.99 (1.52–2.59)	<0.001
Nontoxic goiter (E04)	1.28 (0.91–1.79)	0.158
Disorders of lipoprotein metabolism (E78)	0.89 (0.70–1.12)	0.318
Dorsalgia (M54)	0.83 (0.67–1.03)	0.092
Gastritis and duodenitis (K29)	1.00 (0.72–1.37)	0.983
Sleep disorders (G47)	1.36 (0.97–1.92)	0.075
Chronic ischemic heart disease (I25)	0.97 (0.71–1.31)	0.829
Type 2 diabetes mellitus (E11)	0.41 (0.29–0.57)	<0.001

* Multivariate logistic regression adjusted for all diagnoses listed in the table; *p* < 0.05 is considered statistically significant.

## Data Availability

The original contributions presented in the study are included in the article, further inquiries can be directed to the corresponding author.
